# Refining the Characterization and Outcome of Pathological Complete Responders after Neoadjuvant Chemotherapy for Muscle-Invasive Bladder Cancer: Lessons from the Randomized Phase III VESPER (GETUG-AFU V05) Trial

**DOI:** 10.3390/cancers15061742

**Published:** 2023-03-13

**Authors:** Stéphane Culine, Valentin Harter, Clémentine Krucker, Gwenaelle Gravis, Aude Fléchon, Christine Chevreau, Hakim Mahammedi, Brigitte Laguerre, Aline Guillot, Florence Joly, Jacqueline Fontugne, Yves Allory, Christian Pfister

**Affiliations:** 1 Department of Medical Oncology, Hôpital Saint-Louis, AP-HP, Nord, Université de Paris Cité, Avenue Claude Vellefaux, 75010 Paris, France; 2 North-West Canceropole Data Center, Baclesse Cancer Center, 14000 Caen, France; 3 CNRS, UMR144, Molecular Oncology Team, Equipe Labellisée Ligue Contre le Cancer, PSL Research University, Institut Curie, 75005 Paris, France; 4 Department of Medical Oncology, Paoli-Calmette Institute, 13009 Marseille, France; 5 Department of Medical Oncology, Léon Bérard Cancer Center, 69008 Lyon, France; 6 Department of Medical Oncology, ICR-IUCT Oncopole, 31100 Toulouse, France; 7 Department of Medical Oncology, Jean Perrin Cancer Center, 63011 Clermont-Ferrand, France; 8 Department of Medical Oncology, Eugène Marquis Cancer Center, 35042 Rennes, France; 9 Department of Medical Oncology, Lucien Neuwirth Cancer Institute, 42270 St Priest en Jarez, France; 10 Department of Medical Oncology, Baclesse Cancer Center, 14000 Caen, France; 11 Department of Pathology, Institut Curie, 92210 Saint-Cloud, France; 12 Université Paris-Saclay, UVSQ, 78180 Montigny-le-Bretonneux, France; 13 Department of Urology, Clinical Investigation Center, Inserm 1404, Charles Nicolle University Hospital, 76000 Rouen, France

**Keywords:** muscle-invasive bladder cancer, cisplatin-based chemotherapy, neoadjuvant treatment, pathological complete response

## Abstract

**Simple Summary:**

On the basis of a deep analysis of data collected in a randomized trial of perioperative chemotherapy in muscle-invasive bladder cancer, we refined the characterization and outcome of patients whose cystectomy specimens were pathologically free of cancer (pCR) after neoadjuvant chemotherapy. Nested variant and lymphovascular invasion were identified as adverse predictive factors of pCR. We confirm that these patients portend a better outcome as compared to patients with invasive disease at cystectomy, with a progression-free survival probability three years after pCR on cystectomy of about 85%. A lower creatinine clearance and the delivery of less than four cycles were associated with a higher risk of relapse.

**Abstract:**

Neoadjuvant cisplatin-based chemotherapy (NAC) followed by radical cystectomy and pelvic lymph node dissection is the optimal treatment for patients with muscle-invasive bladder cancer. In recent years, the VESPER trial showed a statistically significant higher progression-free survival with dd-MVAC (dose dense methotrexate, vinblastine, doxorubicin, and cisplatin) compared to GC (gemcitabine and cisplatin). In the present report, we refine the characterization and outcome of patients whose cystectomy specimens were pathologically free of cancer (pathological complete response, pCR). We confirm that these patients portend a better outcome as compared to patients with invasive disease (≥pT1N0) at cystectomy. Nested variant and lymphovascular invasion were identified as adverse predictive factors of pCR. Progression-free survival probability three years after pCR on cystectomy was about 85%, regardless of the NAC regimen. A lower creatinine clearance and the delivery of less than four cycles were associated with a higher risk of relapse. Predicting the efficacy of NAC remains a major challenge. The planned analysis of molecular subtypes in the VESPER trial could help predict which patients may achieve complete response and better outcome.

## 1. Introduction

Radical cystectomy (RC) and extended pelvic lymph node dissection remain the optimal local therapy for patients with non-metastatic muscle-invasive bladder cancer (MIBCa) [1,2]. However, this treatment can usually cure only 50% of patients, suggesting a high frequency of micro-metastases at diagnosis [3,4]. In the 1990s, neoadjuvant cisplatin-based combination chemotherapy (NAC) was developed to treat the potential micrometastatic systemic disease and consequently improve patient outcome. A pivotal trial that used neoadjuvant methotrexate, vinblastine, doxorubicin, and cisplatin (MVAC) demonstrated that three cycles of NAC over 3 months could achieve higher pathological downstaging rates at surgery and better long-term survival [5]. Similar results were reported in a randomized trial that used three cycles of methotrexate, vinblastine, and cisplatin (CMV) as NAC [6]. The demonstration of a survival benefit based on level 1 evidence led to establishing the sequence of NAC followed by RC as a standard of care in patients with MIBCa, providing appropriate conditions for cisplatin administration be met [7,8]. Due to significant toxicities observed with MVAC or CMV regimens, including toxic deaths, alternative combinations such as dose dense MVAC (dd-MVAC) or gemcitabine-cisplatin (GC), were analyzed over the last two decades in prospective studies [9,10,11]. Retrospective studies have reported divergent results as to the respective efficacy of these two regimens [12,13,14].

The French Genitourinary Tumor Group (GETUG) conducted a randomized phase III trial in order to determine the optimal cisplatin-based chemotherapy regimen in the perioperative setting, i.e., dd-MVAC or GC [15]. The analysis of the primary end-point showed a statistically significant higher progression-free survival in the dd-MVAC arm for patients who received NAC [16]. We focus in the present report on patients who achieved a complete pathological response (pCR), i.e., patients whose surgical specimens were pathologically free of cancer (ypT0N0 stage). The main objectives were to consider their initial clinical and pathological characteristics, as well as treatment delivery, in order to refine pathological review of pCR and to compare their outcome to patients who did not achieve pCR.

## 2. Patients and Methods

### 2.1. Patients and Treatments

As previously described, the VESPER trial recruited 500 MIBCa patients from February 2013 to March 2018 in 28 French participating centers after informed signed consent (clinicaltrials.gov identifier NCT01812369) [15]. Key inclusion criteria were histologically confirmed muscle-invasive urothelial carcinoma and disease defined by a cT2, cT3, or cT4a N0 (lymph node ≤10 mm on CT scan) M0 staging for patients receiving NAC (N = 437) or pT3/pT4 or pN+, regardless of pT stage and M0 for patients receiving adjuvant chemotherapy (N = 56). Patients received after randomization either four cycles of the GC regimen (gemcitabine 1250 mg/m^2^ on days 1 and 8 and cisplatin 70 mg/m^2^ on day 1 every 3 weeks) or six cycles of the dd-MVAC regimen (methotrexate 30 mg/m^2^ on day 1, vinblastine 3 mg/m^2^, doxorubicin 30 mg/m^2^, and cisplatin 70 mg/m^2^ on day 2 with G-CSF from day 3 to day 9, every 2 weeks).

### 2.2. Pathological Review

**Figure 1:**
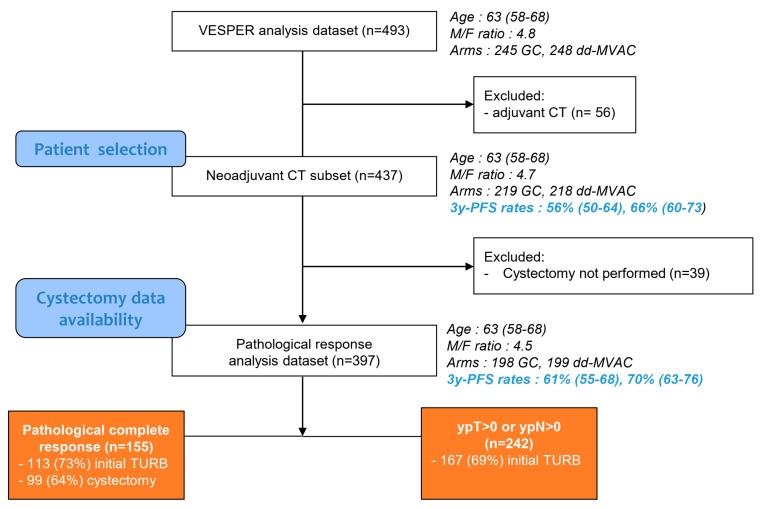
Flow diagram of the GETUG/AFU VESPER V05 trial for analyses of pathological response of patients who received neoadjuvant chemotherapy. CT = chemotherapy.

A central pathological review for both initial transurethral resections of the bladder (TURB) and cystectomy specimens was performed by an expert genito-urinary pathologist (Y.A.). Among the pathological response analysis dataset, 280 (71%) TURB specimens and 99 (64%) cystectomy specimens with pCR were available for pathological review (Figure 1). The following features were assessed: for TURB, histological subtype or presence of a divergence, tumor grade according to the WHO 2016 classification [17], tumor stage, lymphovascular invasion (LVI), perineural invasion, proportion of tumor necrosis, and associated flat carcinoma in situ (CIS); for cystectomy, ypT0N0 status check according to the TNM UICC classification (8th edition), post-chemotherapy regression features (necrosis, presence of muscle dissecting fibrosis, proportion of detrusor thickness impacted by fibrosis, perivesical fat impacted by fibrosis), and post resection granuloma. With the hypothesis that depth of fibrosis might reflect initial tumor staging, depth of fibrosis in the detrusor (T2a under 50%; T2b above 50%) and/or perivesical fat (T3) was evaluated.

### 2.3. Statistical Methods

Regarding descriptive results, qualitative data were reported as frequency and percentage and quantitative data as either means and standard deviation or as median and 95% range, depending on the normal distribution assessment of the variable. Baseline characteristics were compared with Student’s *t*-test, the Wilcoxon–Mann–Whitney test, the chi-squared test, or Fisher’s exact test depending on whether their statistical assumptions were met. For analyses regarding chemotherapy delivery, tests were stratified on the randomization arm. As pathological response is a time-dependent parameter, in order to analyze its relationship with progression-free survival and present easily interpretable results, the definition of this endpoint was slightly modified. In this study, 3-year progression-free survival (hereafter referred to as 3y-PFSc) was defined as the time to the detection of bladder cancer progression or to the patient’s death within 3 years of cystectomy. Survival curves were computed with the Kaplan–Meier method. Corrected Akaike information criterion (AICc) was used to select, step-by-step, the best-fitting Cox proportional hazard models. All statistical analyses were performed with R statistical software v4.0.2 (R Core Team (2020); R: A language and environment for statistical computing; R Foundation for Statistical Computing, Vienna, Austria).

## 3. Results

### 3.1. Baseline Patient Characteristics

Among the 397 patients selected who received NAC, 155 (39%) were free of cancer in RC specimens. Baseline characteristics of patients who achieved a pCR compared to those who did not obtain a pCR are reported in Table 1. Among clinical parameters, only a trend towards a higher body mass index in patients with no residual disease was observed. A pathological review of the initial TURB identified a lesser rate of pCR in patients with the nested histological subtype and in the presence of lymphovascular invasion.

### 3.2. Treatment Delivery

**Figure 2:**
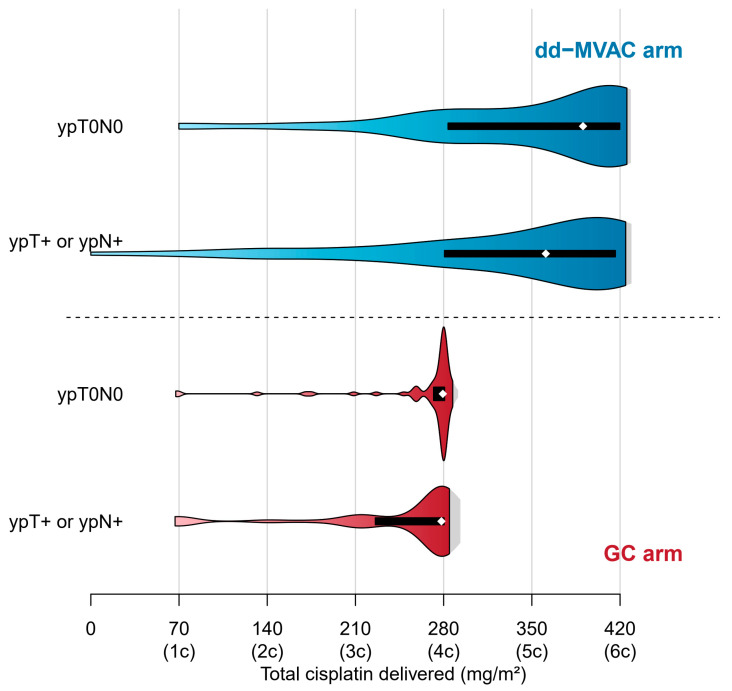
Distribution of total cisplatin dose delivered according to randomization arm and pathological response. Violin plots with Gaussian kernel density estimation representing total cisplatin dose (mg/m²) distribution among patients. The colored areas of the violin represent the 95% confidence interval of the distribution. The black box represents the first and third quartiles’ interval. The white point gives the median. Equivalence between the dose and the number of cures is given on the x-axis.

The median number of cycles was similar in patients with or without pCR. Regarding cisplatin delivery, no difference was observed in the total number of cycles, the number of cycles with a full dose of cisplatin, or in cisplatin dose intensity between both populations. However, the median total dose of cisplatin was higher in patients who achieved a pCR (Table 2). In the dd-MVAC arm, the proportion of patients who achieved a pCR was similar after four cycles (43%), five cycles (38%), or six cycles (45%), while surgery before four cycles led to lower results (29%). In the GC arm, pCR was clearly different when cystectomy was performed before (21%) or after (38%) 4 cycles (Figure 2).

### 3.3. Pathological Characterization of pCR

After the central review of 99 cystectomy specimens, the ypT0N0 status was confirmed in all cases except for two (ypTis and ypT1 stage). No lymphovascular invasion was detected. Necrosis foci without viable tumor cells was observed in five cases. Muscle-dissecting fibrosis was seen in all patients except one. In most patients (89%), fibrosis dissected >50% of the detrusor. Fibrotic changes were observed in the perivesical fat in 28% of patients. Post-resection granulomas were seen in the submucosa and/or the superficial part of the detrusor in 51 (52%) cases.

### 3.4. Patient Outcome

**Figure 3:**
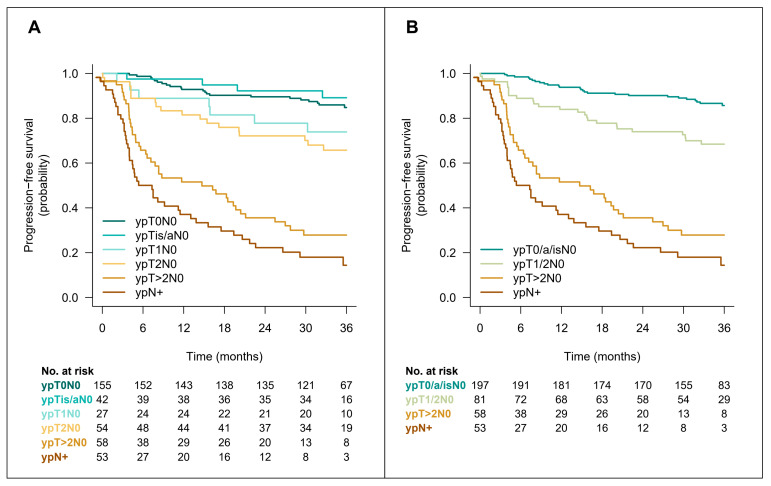
Three-year-progression-free survival Kaplan–Meier curves according to detailed pathological response stage (**A**) or best-fitting grouping after step-by-step optimization of AICc on the Cox proportional hazard model (**B**).

**Figure 4:**
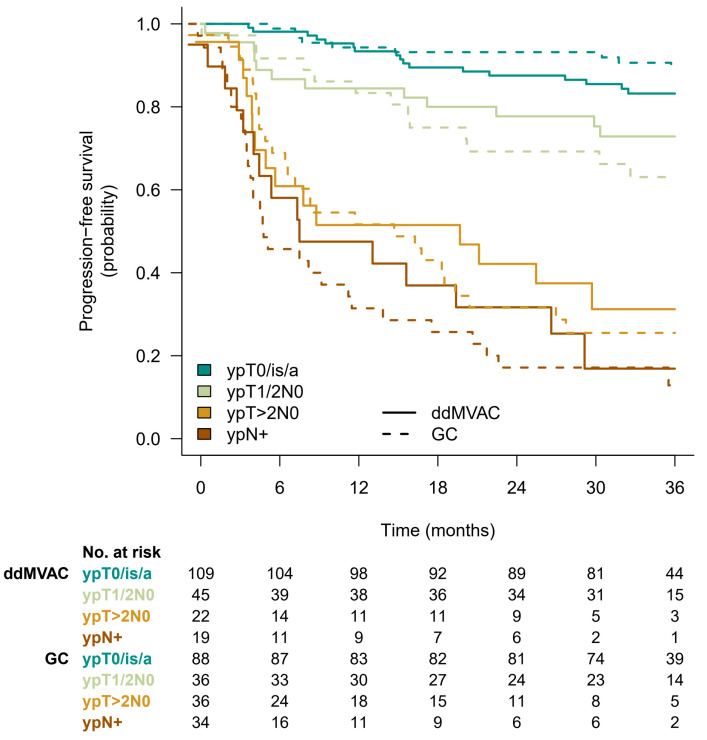
Three-year year-progression-free survival Kaplan–Meier curves according to randomization arm and best-fitting pathological response stage grouping.

Among the 155 patients who were free of disease at cystectomy, the 3y-PFSc rate was 85% (95%CI 79–91) with no difference between the randomization arm (*p* = 0.5). For patients without pCR, the 3y-PFSc rates were 89%, 74%, 66%, 28%, and 14% for patients with ypTa/isN0, ypT1N0, ypT2N0, ypT3N0, and ypN+ disease at cystectomy, respectively. The progression-free survival curves are shown in Figure 3A. Sites of relapse were similar in patients with or without pCR (Table 3). The outcome of patients with pCR was similar to that of patients with noninvasive (ypTa/isN0) disease. This observation was confirmed with optimization of the Cox proportional hazard model adjusted for pathological response. For 3y-PFSc regression, the best-fitting model suggested grouping patients into four classes: ypT0/a/isN0, ypT1/2N0, ypT > 2N0, and ypN+ (Figure 3B). When considering the impact of treatment arm beyond pathological response on 3y-PFSc, there was no evidence of added benefit for dd-MVAC (*p* = 0.7) and no significant interaction with pathological response (Figure 4).

### 3.5. Variables Affecting Outcome of Patients with pCR

Baseline and treatment delivery characteristics were studied in univariate Cox proportional hazard models to determine their potential role in relapse among patients with pCR at RC (Table 4). The only adverse prognostic factors were an elevated neutrophil count, a low creatinine clearance, and less than four cycles of dd-MVAC chemotherapy. Conversely, higher body max index and creatinine clearance was a protective factor against the risk of relapse.

## 4. Discussion

In general oncology, patients who achieve pCR have more favorable outcomes than those who do not. Although a treatment that increases the proportion of patients with pCR should rationally lead to more cured patients, high-quality evidence data suggest that this is not necessarily true [18]. Importantly, pCR reflects the combined effect of both TURB and chemotherapy on the primary tumor. However, the effect of chemotherapy on the potential micrometastatic systemic disease outside the primary tumor is likely to be the primary driver of outcome. Micrometastatic cells may be phenotypically or genotypically different from most cells in the primary tumor and may not respond to chemotherapy, even if pCR is achieved in the primary tumor site [19].

In MIBCa, cumulative evidence has been reported for a better survival in patients who achieved pCR compared to non-responder patients [5,20,21,22,23,24]. In the randomized phase III trial using neoadjuvant MVAC as experimental arm, the 5-year survival of patients who achieved pCR at RC was twice as high irrespective of treatment arms, i.e., with or without NAC [5]. In contrast, the Nordic Urothelial Cancer Group reported a better survival limited to patients who achieved a pCR following NAC [22]. Another study suggested that a decrease in at least one stage in downstaging from clinical to pathologic stage was associated with improved survival [24]. We also observed a better progression-free survival for patients with pCR. The benefit was not different for patients with non-invasive disease (pTa/pTis pN0), while patients with pT1pN0 residual disease had a poorer outcome, very similar to patients with pT2pN0 residual disease. There are no data supporting pCR as a valid surrogate endpoint for survival outcome. However, pCR has been used as a primary endpoint for assessing efficacy of neoadjuvant therapies in retrospective and prospective phase II studies over the last two decades. In the VESPER trial, despite similar pCR rates in both arms (42% in the dd-MVAC arm and 36% in the GC arm), the 3-year PFS was clearly improved with the dd-MVAC regimen [16,25], suggesting a greater effect on micrometastatic systemic disease. The post-NAC pathological organ-confined disease (<ypT3N0) rate was statistically different between the two arms (77% in the dd-MVAC arm and 63% in the GC arm) [12]. According to these objective data, organ-confined disease might be a better surrogate than pCR for predicting outcome.

Despite a better outcome for patients with pCR, the 3y-PFSc rate after RC remains at about 85%, regardless of the NAC regimen in this population. These results are in accordance with previous prospective and retrospective reports [5,20,21,22,23,24], suggesting a lesser efficacy of NAC on micrometastatic disease than on primary tumor in a minority of patients. The sites and time to relapse were not different from those observed in patients who did not achieve a pCR. The initial pathological features were not associated with the risk of relapse in pCR patients. In particular, we did not identify lymphovascular invasion or carcinoma in situ as adverse prognostic factors, in contrast to others [20]. Keeping in mind that the primary tumor pathological stage results from the effect of both TURB and NAC, we tried to refine the pathological assessment of pCR on cystectomy specimens. We hypothesized that granulomas in the submucosa and/or the superficial part of the detrusor could be related to TURB, while the depth of fibrosis in the detrusor might reflect the initial tumor stage and the efficacy of chemotherapy. We found no impact of these features on outcome in pCR patients. However, these results are limited since the central review could be conducted in only 64% of cystectomy specimens. The only adverse prognostic factors were an elevated neutrophil count, a low creatinine clearance, and a number of less than four cycles of dd-MVAC. An initial elevated neutrophil count may indicate increased inflammation and a poor antitumor response, as suggested in previous reports focusing on the prognostic value of the neutrophil-to-lymphocyte ratio in MIBC [26].

Predicting the efficacy of NAC remains a major challenge. We previously reported the potential impact of body mass index on pCR without being able to provide an explanation for this observation [27]. Among TURB pathological features, the nested variant as well as LVI were associated with a lower probability of pCR. Variant histological subtypes of urothelial carcinoma are known to exhibit aggressive biological behavior. Although optimal management remains to be defined, NAC and radical cystectomy with pelvic lymph node dissection remains the mainstay of treatment [28,29]. The nested subtype is a rare divergent differentiation of urothelial carcinoma [30]. It belongs to the luminal molecular subtype but seems characterized by specific mutations in Wnt and inflammatory pathways [31]. LVI and necrosis have been described as adverse prognostic features in the cystectomy specimens [32,33,34]. Our study also argues for their negative impact on achieving pCR after NAC. Finally, our results suggest that the higher the cumulative dose of cisplatin, the greater the likelihood of achieving pCR, whatever the arm of the VESPER trial. This is in accordance with previous data arguing radical cystectomy (RC) and extended pelvic lymph node dissection remains the optimal local therapy for patients with non-metastatic muscle-invasive bladder cancer (MIBCa) for a lesser chance of obtaining a pCR when less than four cycles of GC or dd-MVAC are delivered [27].

Major limitations of our study include the lack of exhaustiveness of available specimens for central pathological review and the lack of data regarding additional tumor characteristics (number, size, complete resection) at initial TURB. In daily practice, the sequence of NAC followed by RC remains the standard of care in patients with MIBCa, irrespective of the presence of histological variants and lymphovascular invasion. A minimum of four cycles of cisplatin-based NAC is mandatory to achieve optimal progression-free survival.

## 5. Conclusions

Complementary data from the VESPER trial confirmed that patients who are free of disease after NAC portend a better outcome as compared to patients with invasive disease (≥pT1N0) at RC. However, prognosis of patients with pCR was not different from that of patients with noninvasive (pTa/isN0) disease. Nested histological subtype and LVI were adverse predictive factors of pCR. Progression-free survival probability three years after a pCR on cystectomy was about 85%, regardless of the NAC regimen. A lower creatinine clearance and the delivery of less than four cycles of NAC were associated with the risk of relapse. The planned analysis of molecular subtypes in the VESPER trial could help predict which patients may achieve complete response.

## Figures and Tables

**Table 1:** Baseline patients’ characteristics according to pathological responses at cystectomy.

Variable	ypT0N0 (N = 155)	ypT > 0 or ypN > 0 (N = 242)	*p*-Value
Age, years, No (%)			
<60	47 (30)	83 (34)	0.6
60–69	78 (50)	120 (50)	
≥70	30 (20)	39 (16)	
Median (95% range)	64 (58–68)	63 (58–68)	0.2
Sex, No (%)			
Male	126 (81)	199 (82)	>0.9
Female	29 (19)	43 (18)	
Body mass index, kg/m^2^, No (%)			
<18.5	4 (2.6)	8 (3.3)	0.046
18.5–<25	41 (26)	96 (40)	
25–<30	74 (48)	90 (37)	
≥30	36 (23)	48 (20)	
Median (95% range)	26.8 (24.4–29.6)	25.8 (22.9–29.3)	0.088
Body surface area (m^2^)			
Median (95% range)	1.9 (1.8–2.1)	1.9 (1.8–2.1)	0.2
Medical history, No (%)			
Tobacco	121 (82)	202 (86)	0.4
Hypertension	62 (42)	98 (42)	>0.9
Coronary artery disease	7 (4.5)	5 (2.1)	0.3
Diabetes	7 (4.5)	8 (3.3)	0.4
Performance status, No (%)			
0	113 (73)	168 (69)	0.8
1	41 (26)	72 (30)	
Unknown	1 (0.6)	2 (0.8)	
Hemoglobin (g/dL)			
Median (95% range)	14.2 (13.2–15)	14.1 (13.2–15.1)	0.4
Alkaline phosphatases (UI/L)			
Median (95% range)	70.5 (59–82.8)	71.5 (60.2–88)	0.2
Serum creatinine (mg/L)			
Median (95% range)	9 (8–10)	9 (8–11)	0.7
Creatinine clearance (mL/min), No (%)			
50–59	9 (5.8)	27 (11)	0.19
60–89	68 (44)	106 (44)	
≥90	78 (50)	109 (45)	
Median (95% range)	90 (73–107)	87 (71–102)	0.17
Initial clinical stage			
T2a	102 (66)	152(63)	>0.9
T2b	41 (26)	73 (30)	
T3a	4 (2.6)	6 (2.5)	
T3b	4 (2.6)	4 (1.7)	
T4a	4 (2.6)	7 (2.9)	
Histology at baseline, No (%)			
Central review	113 (73)	167 (69)	
Pure urothelial carcinoma	50 (44)	64 (38)	0.4
Urothelial carcinoma with divergence or subtypes > 10%	63 (56)	103 (62)	
Squamous	22 (19)	38 (23)	0.6
Glandular	5 (4.4)	12 (7.2)	0.5
Micropapillary	16 (14)	17 (10)	0.4
Nested	6 (5.3)	23 (14)	0.038
Sarcomatoid	9 (8.0)	8 (4.8)	0.4
Concomitant carcinoma in situ	49 (43)	82 (49)	0.4
Lymphovascular invasion	36 (32)	75 (45)	0.039
Perineural invasion	7 (6.2)	16 (9.6)	0.4
Necrosis > 10%	13 (12)	34 (20)	0.075

**Table 2:** Treatment delivery and pathological responses at cystectomy.

	pT0pN0		pT > 0 or p > N0		*p*-Value
	GC	dd-MVAC	GC	dd-MVAC	
	(N = 71)	(N = 84)	(N = 127)	(N = 115)	
Number of cycles of chemotherapy Median (range)	4 (1–4)	6 (1–6)	4 (1–4)	6 (0–6)	
0	0	0	0	1	0.4
1	2	3	11	4	
2	1	1	3	5	
3	3	3	9	7	
4	65 (92%)	12 (14%)		16 (14%)	
5		8 (9.5%)		13 (11%)	
6		57 (68%)		69 (60%)	
Total number of cycles	273	444	460	578	
Cisplatin delivery Number of cycles with cisplatin	273	441	460	575	
Number of cycles with full doses of cisplatin	260 (95%)	383 (87%)	421 (92%)	485 (84%)	0.062
Total dose (mg) Median (95% range)	532 (215–603)	720 (166–877)	518 (128–607)	689 (168–903)	0.028
Dose intensity (mg/m^2^/week) Median (95% range)	22.6 (18.7–24.2)	32.1 (21.3–35.6)	23.0 (16.3–24.0)	31.6 (21.8–35.8)	>0.9
Relative dose intensity	97%	92%	99%	90%	
Time interval between randomization and surgery (days) Median (95% range)	121 (90–149)	121 (62–179)	111 (48–164)	118 (62–211)	0.034
Time interval between last course of chemotherapy and surgery (days) Median (95% range)	51 (28.5–88.5)	53.5 (27–93)	47 (23–99.5)	50 (23–145)	0.4

**Table 3:** Site of relapse according to pathological responses.

Relapses	ypT0N0 (n = 155)	ypTa/isN0 (n = 42)	ypT1N0 (n = 27)	ypT2N0 (n = 54)	ypT>2N0 (n = 60)	ypN+ (n = 55)
Number of patients (%)	16 (10)	3 (7.1)	5 (18)	15 (28)	38 (63)	43 (78)
Sites						
Loco-regional (pelvis)	3	1	2	6	12	18
Metastatic	13	2	3	9	26	25
Lung	2	1	0	2	12	9
Liver	4	0	1	2	7	11
Bone	6	1	0	3	8	9
Other	1	0	2	2	0	0

**Table 4:** Three-year-progression-free survival covariables of patients with complete pathological response. Univariate hazard ratio on Cox proportional hazard models.

Variable	n	HR (CI 95%)	*p*-Value
Age, years			
<60	47	-	
60–69	78	1.38 (0.48–3.99)	0.5
≥70	30	2.02 (0.62–6.61)	0.2
Sex			
Male	126	-	
Female	29	0.43 (0.10–1.85)	0.3
Body mass index, kg/m^2^			
<18.5	4	1.42 (0.18–11.1)	0.7
18.5–<25	41	-	
25–<30	74	0.36 (0.14–0.96)	0.040
≥30	36	0.43 (0.14–1.38)	0.16
Body surface area (m^2^)			
		0.15 (0.020–1.13)	0.066
Medical history			
Tobacco	121	0.92 (0.34–2.50)	0.9
Hypertension	62	1.62 (0.70–3.74)	0.3
Coronary artery disease	13	1.71 (0.51–5.78)	0.4
Diabetes	7	2.48 (0.58–10.6)	0.2
Performance status			
0	113	-	
1	41	1.14 (0.45–2.91)	0.8
Biology at baseline			
Hemoglobin			
≥12 g/dL	142	-	
<12 g/dL	13	2.69 (0.91–7.95)	0.074
Neutrophil polynuclear cells			
≤7000/mm^3^	139	-	
>7000/mm^3^	16	2.83 (1.04–7.67)	0.041
Creatinine clearance (mL/min)			
≥90	78	-	
60–89	68	1.33 (0.54–3.27)	0.5
50–59	9	4.16 (1.12–15.4)	0.033
Histology at baseline			
Central review	113		
Urothelial carcinoma with divergence or subtypes > 10%	63	1.24 (0.44–3.47)	0.7
Concomitant carcinoma in situ	49	1.11 (0.40–3.06)	0.8
Lymphovascular invasion	36	1.86 (0.67–5.13)	0.2
Perineural invasion	7	1.07 (0.14–8.14)	>0.9
Necrosis > 10%	62	0.78 (0.28–2.15)	0.6
Treatment delivery (multivariate model)			
Randomization arm			
GC	71	-	
ddMVAC	84	0.73 (0.19–2.84)	0.6
Cisplatin dose/cycle equivalence			
for GC arm			
≥270 mg/m²/4 cycles	55	-	
<270 mg/m²/<4 cycles	16	0.49 (0.06–4.00)	0.5
for ddMVAC arm			
≥410 mg/m²/6 cycles	32	-	
340 to 410 mg/m²/5 cycles	22	1.98 (0.44–8.85)	0.4
270 to 340 mg/m²/4 cycles	17	1.27 (0.21–7.59)	0.8
<270 mg/m²/< 4 cycles	13	5.38 (1.28–22.54)	0.021
Clinical stage inferred by fibrosis on cystectomy (N = 99)			
T2a	17	-	
T2b	54	1.63 (0.36–7.45)	0.5
T3	28	1.24 (0.23–6.77)	0.8

## Data Availability

Data are generated by the data center and are the property by the sponsor according to French law.
